# Dataset for the development of a diagnostic schedule for a defective LC-195V5 CNC milling machine at FUTA central workshop

**DOI:** 10.1016/j.dib.2018.10.160

**Published:** 2018-11-03

**Authors:** O.O. Agboola, P.P. Ikubanni, B.T. Ogunsemi, R.A. Ibikunle, A.A. Adediran, B. Kareem, B.O. Akinnuli, C.O. Osueke

**Affiliations:** a Department of Mechanical Engineering, Landmark University, PMB 1001 Omu-Aran, Kwara State, Nigeria; b Department of Mechanical Engineering, Federal University of Technology Akure (FUTA), Ondo State, Nigeria

**Keywords:** Diagnostic schedule, CNC machine, Defective, PERT, Scheduling

## Abstract

The dataset represented in this article describe a diagnostic schedule for a defective LC-195V5 CNC milling machine using PERT. The efficiency of the technicians who repaired the CNC machine tools was measured based on fault location within the shortest possible time. A diagnostic schedule was developed which showed the sequential means of troubleshooting within a possible shortest time. Two approaches were employed. Forward Pass (FP), which involved the diagnosis from electrical parts through Computer (CNC) to mechanical components and Backward Pass (BP) which involved the diagnosis from computer component through electrical parts to mechanical parts. Three different levels of expertise (trials) were used for each of the mode of diagnosis and the time to diagnose each component part was recorded. Two separate PERT network diagrams were drawn based on the inter-relationship of the component parts of the machine and their Critical Paths were determined.

**Specifications table**

**Table:** 

Subject area	Mechanical Engineering
More specific subject area	Industrial Engineering, Production Engineering
Type of data	Tables, Figures
How data was acquired	Stopwatch, Oscilloscope, Signal generator, multi-meter, line tester, RCL tester, Neon tester, etc. were employed through the usage of Forward pass and Backward pass experiments.
Data format	Raw, processed and analysed
Experimental factors	Having studied the principle of operation of the machine using the operation manual, the machine was tested to ascertain its effectiveness. The machine was found to be faulty and then diagnosis steps were taking to detect the faults. Average expected time for both experiment were determined in which optimistic time, pessimistic time and most likely time were obtained.
Experimental features	Computational Analysis. Optimistic time, pessimistic time and most likely time data obtained from the average/expected time for both the Forward and Backward pass experiments.
Data source location	Diagnostic schedule data was obtained from the Mechanical central workshop of Federal University of Technology, Akure. (**Latitude:** 7°12׳18.04" N, **Longitude:** 5°11׳15.58" E)
Data accessibility	All the data are within this article.
Related research article	Agboola OO, Kareem B, Akinnuli BO. Development of a diagnostic schedule for a defective LC-195V5 CNC milling machine using PERT. Leonardo Electronic Journal of Practices and Technologies, 2016, 15(28):107–118 [1]

**Value of the data**

• The dataset can be used to show the sequence of diagnosing a defective LC-195V5 milling machine and the time take to diagnose each component part.
• The data can be used to evaluate the two possible routes of diagnosis through the setting of Experiments I and II.
• The reported data can be useful for relating information on the relationship between sequence of diagnosis and time taken for the overall diagnosis of the machine as a whole.

## 1 Data

**Fig. 1:**
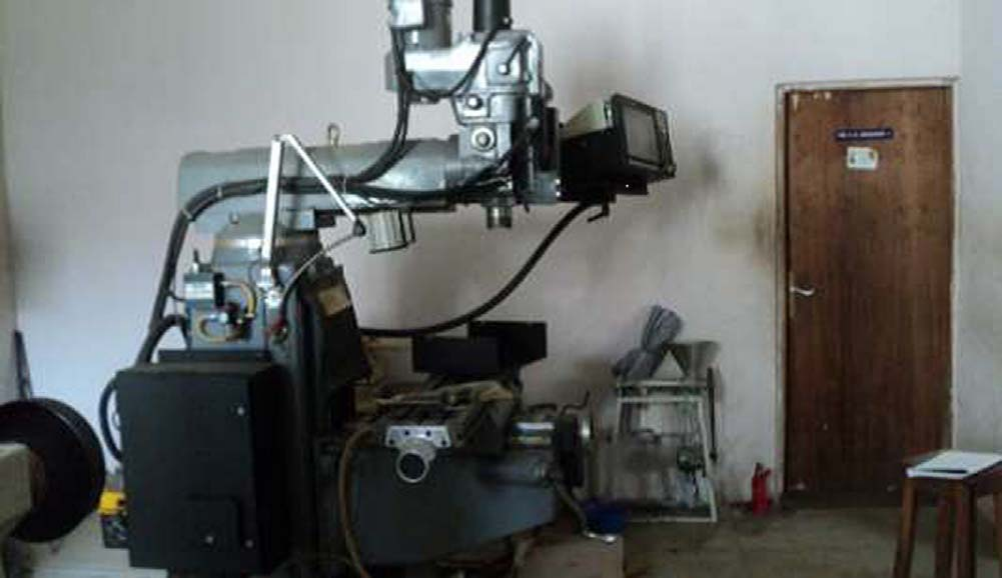
Defective LC-195V5 Milling machine.

**Fig. 2:**
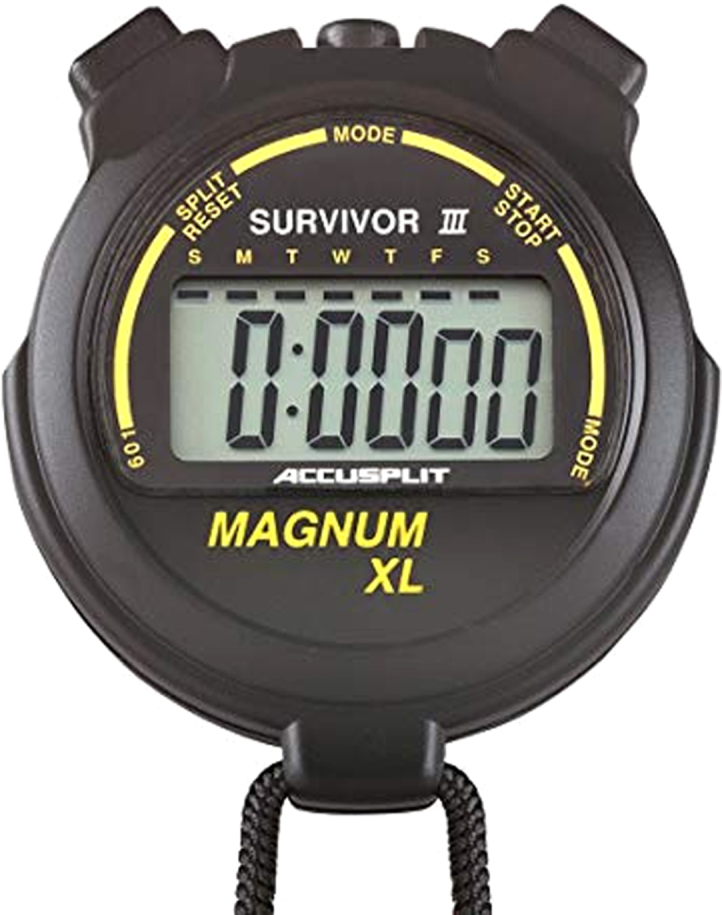
Stopwatch.

**Fig. 3:**
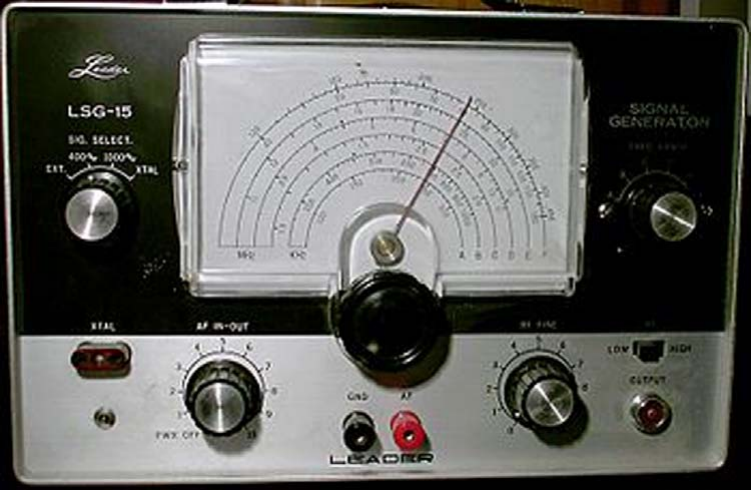
Signal generator.

**Fig. 4:**
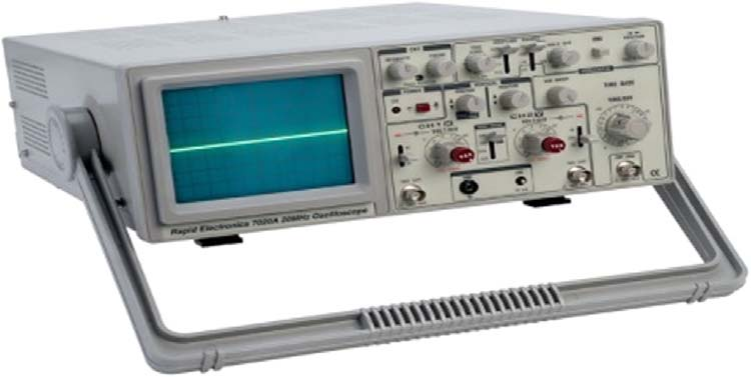
Oscilloscope.

**Fig. 5:**
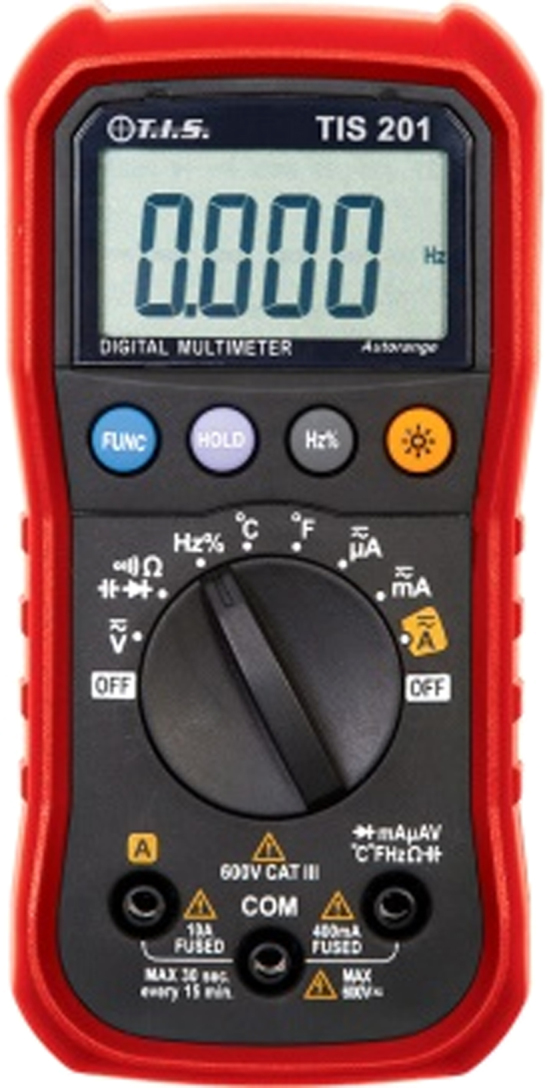
Multimeter.

**Fig. 6:**
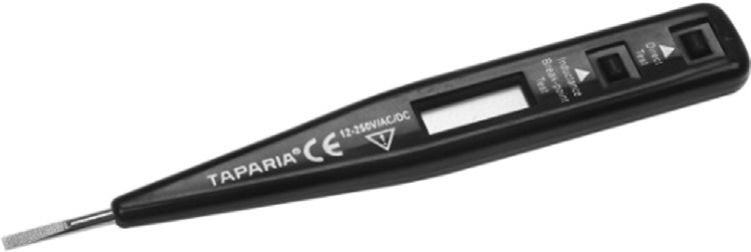
Line tester.

**Fig. 7:**
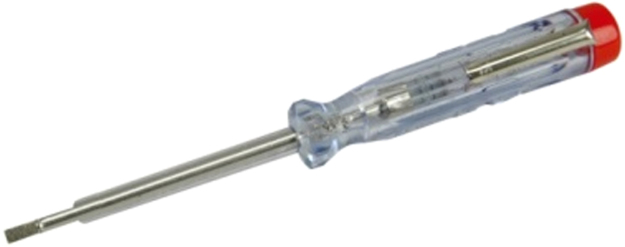
Neon tester.

The dataset presented in this article are experimental results obtained from diagnosis of each component part of the CNC milling machine. The picture of the machine is as shown in Fig. 1. Stopwatch was used to record the time taken to diagnose each component. Other equipment utilized in obtaining this data are as shown in Fig. 2, Fig. 3, Fig. 4, Fig. 5, Fig. 6, Fig. 7. Time to diagnose each component part using Experiments I and II are as shown Table 1, Table 2, respectively. The average/expected time and standard deviation computed for Experiments I and II are as shown in Table 3, Table 4, respectively.

**Table 1:** Experiment I (forward pass)-diagnosis from electrical components.

S/N	Diagnosed parts	Time duration (seconds)		
		1st Trial ‘a’	2nd Trial ‘m’	3rd Trial ‘b’
1	Testing and diagnosis of the electrical mains	698	720	732
2	Checking of the electrical cabinet of the machine	1,265	1,306	1,329
3	Checking, testing and the diagnosis of the spindle motor	1,226	1,265	1,287
4	Checking, testing and the diagnosis of the table motor	1,043	1,077	1,096
5	Testing of the actuator	1,037	1,070	1,089
6	Diagnosis of the processor	1,103	1,141	1,160
7	Checking, testing and diagnosis of the power pack	1,618	1,671	1,701
8	Checking of the CNC console	1,229	1,259	1,282
9	Testing of the coolant sensor	2,261	2,341	2,381
10	Mechanical check of the machine	1,977	2,037	2,076

**Table 2:** Experiment II (backward pass)-diagnosis from computer components.

S/N	Diagnosed parts	Time duration (seconds)		
		1st Trial ‘a’	2nd Trial ‘m’	3rd Trial ‘b’
1	Checking and diagnosis of the CNC console	1,867	1,928	1,962
2	Testing and diagnosis of the processor	1,511	1,563	1,589
3	Checking and testing of the actuator	1,162	1,202	1,223
4	Checking, testing, diagnosis and the repair of the power pack	2,799	2,897	2,948
5	Checking of the electrical cabinet	2,439	2,520	2,567
6	Testing and checking of the spindle motor	1,561	1,613	1,648
7	Testing and checking of the table motor	1,273	1,331	1,356
8	Diagnosis for the mechanical fault	2,046	2,099	2,145

**Table 3:** Average/expected time for experiment I (forward pass).

**Diagnosed parts**	**Time “a”**	**Time “m”**	**Time “b”**	**Expected**	**Standard deviation**
				**Time (s)**	
Testing and diagnosis of the electrical mains	698	720	732	718.33	5.67
Checking of the electrical cabinet of the machine	1,265	1,306	1,329	1,303	10.67
Checking, testing and the diagnosis of the spindle motor	1,226	1,265	1,287	1,262.17	10.17
Checking, testing and the diagnosis of the table motor	1,043	1,077	1,096	1,074.5	8.83
Testing of the actuator	1,037	1,070	1,089	1,067.67	8.67
Diagnosis of the processor	1,103	1,141	1,160	1,137.83	9.5
Checking, testing and diagnosis of the power pack	1,618	1,671	1,701	1,667.17	13.83
Checking of the CNC console	1,229	1,259	1,282	1,257.83	8.82
Testing of the coolant sensor	2,261	2,341	2,381	2,334.33	20
Mechanical check of the machine	1,977	2,037	2,076	2,033.5	16.5

**Table 4:** Average/expected time for experiment II (backward pass).

**Diagnosed parts**	**Time “a”**	**Time “m”**	**Time “b”**	**Expected time (s)**	**Standard deviation**
Testing and diagnosis of the processor	1,511	1,563	1,589	1,558.67	13
Checking and testing of the actuator	1,162	1,202	1,223	1,198.83	10.17
Checking, testing, diagnosis and the repair of the power pack	2,799	2,897	2,948	2,889.17	24.83
Checking of the electrical cabinet	2,439	2,520	2,567	2,514.33	21.33
Testing and checking of the spindle motor	1,561	1,613	1,648	1,610.2	14.5
Testing and checking of the table motor	1,273	1,331	1,356	1,325.5	13.83
Diagnosis for the mechanical fault	2,046	2,099	2,145	2,097.83	16.5

**Fig. 8:**
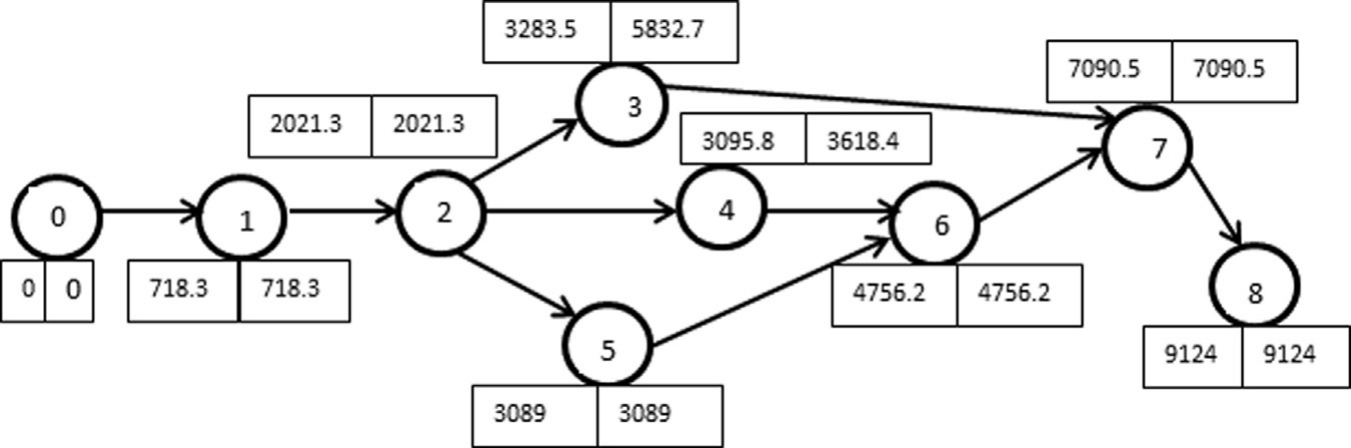
Network diagram for Experiment I.

**Fig. 9:**
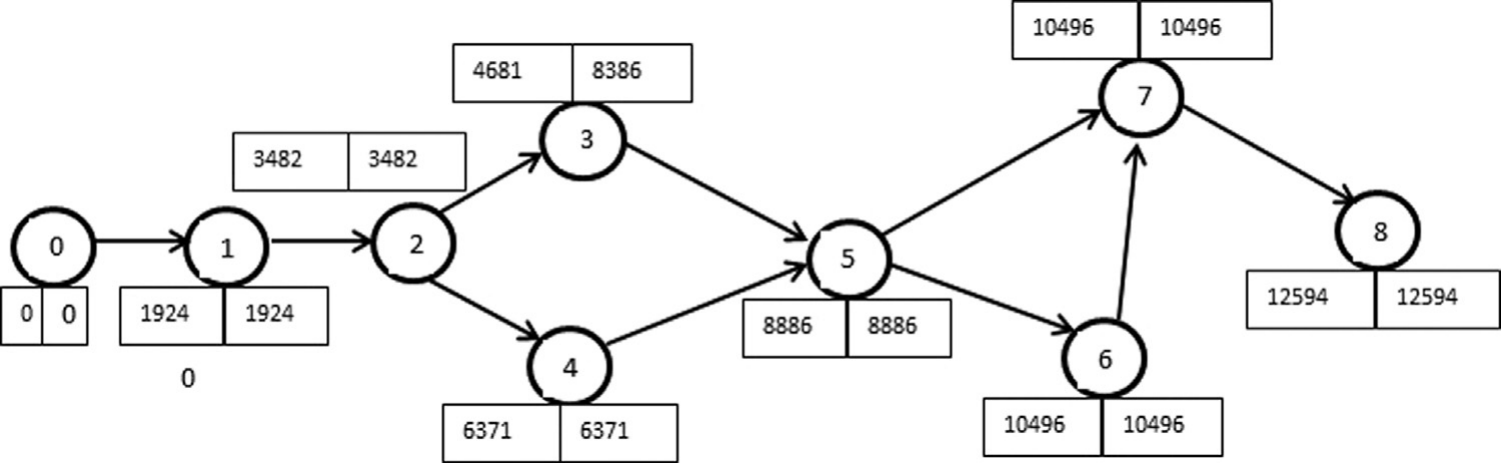
Network diagram for Experiment II.

Fig. 8, Fig. 9 show the network diagram generated from Table 3, Table 4. Analytical tables used in estimating the critical path are as displayed in Table 5, Table 6 for Experiment I and II, respectively.

**Table 5:** Analytical table for estimating critical path for Experiment I.

**Activity**	**Duration (s)**	**Earliest start (ES)**	**Earliest finish (EF)**	**Latest start (LS)**	**Latest finish (LF)**	**Slack**	**Standard deviation**
0–1	718.33	0	718.3	0	718.3	0	5.7
1–2	1,303	718.3	2,021.3	718.33	2,021.3	0	10.7
2–3	1,262.17	2,021.3	3,283.5	4,570.5	5,832.7	2,549.2	10.2
2–4	1,074.5	2,021.3	3,095.8	2,543.9	3,618.4	522.6	8.8
2–5	1,067.67	2,021.3	3,089	2,021.3	3,089	0	8.7
4–6	1,137.83	3,095.8	4,233.6	3,618.4	4,756.2	522.6	9.5
5–6	1,667.17	3,089	4,756.2	3,089	4,756.2	0	13.8
3–7	1,257.83	3,283.5	4,541.3	5,832.7	7,090.5	2,549.2	8.8
6–7	2,334.33	4,756.2	7,090.5	4,756.2	7,090.5	0	20
7–8	2,033.5	7,090.5	9,124	7,090.5	9,124	0	16.5

**Table 6:** Analytical table for estimating critical path for Experiment II.

**Activity**	**Duration (s)**	**Earliest start (ES)**	**Earliest finish (EF)**	**Latest start (LS)**	**Latest finish (LF)**	**Slack**	**Standard deviation**
0–1	1,923.5	0	1,923.5	0	1,923.5	0	15.83
1–2	1,558.67	1,923.5	3,482.2	1,923.5	3,482.2	0	13
2–3	1,198.83	3,482.2	4,681	7,686.9	8,885.7	4,204.7	10.17
2–4	2,889.17	3,482.2	6,371.4	3,482.2	6,371.4	0	24.83
4–5	2,514.33	6,371.4	8,885.7	6,371.4	8,885.7	0	21.33
3–5	0	4,681	8,885.7	4681	8,885.7	0	0
(Dummy)							
5–6	1,610.2	8,885.7	10,495.9	8,885.7	10,495.9	0	14.5
5–7	1,325.5	8,885.7	10,211.2	9,170.4	10,495.9	284.7	13.83
6–7	0	10,495.9	10,495.9	10,495.9	10,495.9	0	0
(Dummy)							
7–8	2,097.83	10,495.9	12,593.7	10,495.9	12,593.7	0	16.5

## 2 Experimental design, materials, and methods

The defective LC-195V5 milling machine is owned by the Federal University of Technology Akure, Nigeria. Two routes or methods of diagnosing tagged Experiment I and Experiment II were used. In Experiment I, the diagnosing was performed from Electrical parts through computer parts to mechanical components. Experiment II on the other hand involves diagnosing from computer components through electrical components to mechanical parts. Diagnostic exercise followed the prescribed procedures [2].

Stopwatch was used to record the time taken to diagnose each component part. For reliability of data, three experts were used in diagnosing the parts and time spent by each one for both experiments were recorded as optimistic time “a”, pessimistic time “b” and mostly likely time “m”.

Average/expected time ($T_{E}$) was computed using Eq. (1) and standard deviation (σ) was obtained using Eq. (2)

(1) $$T_{E}=\frac{a+4m+b}{6}$$

(2) $$\sigma =\frac{\left(b-a\right)}{6}$$

Signal generator was used for generating electronic signals (repeating and non-repeating signals); Oscilloscope was used to display and analyse waveform of electronic signals; multimeter was used measure voltage, current and resistance; line tester was used to test phase/live or positive conductor; RCL tester was used to simultaneously detect resistance, capacitance and inductance; while neon tester was for electrical testing.
