# Supplementary material for: Dataset for the development of a diagnostic schedule for a defective LC-195V5 CNC milling machine at FUTA central workshop
Source: Data Brief. 2018 Nov 3;21:1496–503. doi: 10.1016/j.dib.2018.10.160 (PMC6258252; doi:10.1016/j.dib.2018.10.160)
Supplement: Supplementary file 2 — Supplementary material [file mmc2.pdf]

LANDMARK UNIVERSITY
KM 4, IPETU ROAD, PMB 1001, OMU-ARAN, KWARA STATE, NIGERIA
www.lmu.edu.ng

RESEARCH OFFICE

Dr. Adebesin, B.O.
Chairman Research Board
+2348058051253
lucerd@lmu.edu.ng

Ref: LUCERD/2018/09/105 *Date: 4th October 2018*

The Editor,
Data in Brief.

INSTITUTIONAL LETTER OF SUPPORT IN RESPECT OF A MANUSCRIPT SUBMITTED TO DATA IN BRIEF

I write in respect of a manuscript entitled **"Dataset for the Development of a Diagnostic Schedule for a Defective LC-195V5 CNC Milling Machine at FUTA Central Workshop**" with manuscript number ***DIB-D-18-02146***; authored by Agboola et al., and returned to Authors for confirmation from the University Management, in support of the dataset used for the work.

I wish to state that the data was sourced from Central Workshop of the Federal University of Technology Akure (FUTA), Nigeria their submission are original and authentic.

Kind regards

Dr. B. O. Adebesin.
Chair., University Research Board.
